# The Role of Eosinophilic Cationic Protein in Inflammatory Bowel Disease

**DOI:** 10.7759/cureus.93934

**Published:** 2025-10-06

**Authors:** Paul Grama, Tamás Ilyés, Naomi A Ciurea, Simona M Bataga

**Affiliations:** 1 1st Internal Medicine Department, George Emil Palade University of Medicine, Pharmacy, Science and Technology of Târgu Mureș, Târgu Mureș, ROU; 2 Department of Gastroenterology, Târgu Mureș Emergency Clinical County Hospital, Târgu Mureș, ROU; 3 Doctoral School, George Emil Palade University of Medicine, Pharmacy, Science and Technology of Târgu Mureș, Târgu Mureș, ROU; 4 Department of Medical Biochemistry, “Iuliu Hațieganu” University of Medicine and Pharmacy, Cluj-Napoca, ROU

**Keywords:** biomarker, crohn’s disease, eosinophilic cationic protein, eosinophils, inflammatory bowel disease, ulcerative colitis

## Abstract

Introduction

Eosinophils contribute to inflammatory bowel disease (IBD) pathogenesis by infiltrating the intestinal mucosa and releasing pro-inflammatory mediators, including eosinophilic cationic protein (ECP). Although biomarkers like C-reactive protein and fecal calprotectin commonly assess IBD activity, the clinical utility of eosinophil-derived markers like ECP is unclear. Our aims were to compare serum ECP across ulcerative colitis (UC), Crohn’s disease, and healthy controls, with the prespecified primary contrast UC vs. controls. Our secondary objectives were to examine associations between ECP and disease activity (CDAI, MAYO scores) and quality of life, and explanatory objectives were to assess correlations with white blood cells and neutrophil and eosinophil counts and explore UC-control discrimination.

Methods

We conducted an observational study including 50 IBD patients (20 Crohn’s disease patients, 30 UC patients) and 32 healthy controls. Clinical data recorded included disease duration, Crohn’s Disease Activity Index (CDAI), Mayo score for UC, and quality of life (IBDQ questionnaire). Serum ECP (enzyme immunoassay), eosinophil counts, and routine blood tests were measured. Statistical analysis employed Kruskal-Wallis with Dunn’s post-hoc tests, Mann-Whitney U tests, and Spearman’s correlation. Ethical approval and informed consent were obtained.

Results

Median serum ECP differed significantly among controls, Crohn’s, and UC patients (16,807 (13,007-24,314) pg/mL vs 18,496 (12,261-28,231) vs 24,224 (17,335-32,997); p=0.03). UC patients had significantly higher ECP than controls (p=0.008), while the increase in Crohn’s disease was not significant (p=0.418). ECP was higher in UC compared to Crohn’s disease, though this difference did not reach statistical significance (p=0.128). No significant differences emerged between Crohn’s disease and UC in total leukocyte count, eosinophil count, disease duration, or Inflammatory Bowel Disease Questionnaire (IBDQ) scores (all p>0.1). Serum ECP positively correlated with total leukocyte (r=0.279, p=0.049) and neutrophil counts (r=0.298, p=0.036) across IBD patients. No significant correlations existed between ECP and patient age, disease duration, CDAI, Mayo score, IBDQ, or eosinophil count (all p>0.1).

Conclusion

In this cross-sectional cohort, serum ECP was higher in UC vs controls and showed only modest associations with leukocytosis, while not tracking CDAI, partial Mayo, or IBDQ. These findings are hypothesis-generating and consistent with eosinophil involvement; they do not demonstrate mechanism or clinical utility and require prospective validation.

## Introduction

Inflammatory bowel diseases (IBD) (Crohn’s disease and ulcerative colitis (UC)) are characterized by chronic intestinal inflammation with involvement of various immune cells. The pathogenesis of IBD includes a complex interplay between genetic susceptibility, immune system dysregulation and external environmental factors. While neutrophils and lymphocytes are well-known mediators of acute intestinal inflammation, eosinophils are also increasingly recognized as important immune cells in the gut mucosa of patients [1-3]. Activated eosinophils release cytotoxic granule proteins, including eosinophilic cationic protein (ECP), major basic protein, and eosinophil peroxidase, which can induce tissue damage and modulate inflammation. In active IBD, elevated levels of eosinophil granule proteins have been found in affected intestinal tissues and fluids, implicating eosinophils in the inflammatory process. ECP can injure cell membranes and is considered one of the key eosinophil-derived mediators involved in local gut inflammation [4]. Evidence suggests that eosinophils are activated in response to the inflammatory milieu characteristic of IBD [5]. There has also been put forward the hypothesis that eosinophil-predominant inflammation in IBD might be associated with a reduced risk of disease flares and hospitalization [1]. However, the clinical significance of eosinophil activation in IBD, and specifically whether eosinophil-derived proteins like ECP correlate with disease activity or outcomes, remains unclear.

Current noninvasive biomarkers for IBD activity mainly put accent on neutrophil-mediated inflammation; for example, serum C-reactive protein and fecal calprotectin are widely used to gauge disease activity and mucosal healing [6]. However, these markers have limitations; CRP is not always elevated in active IBD, and fecal calprotectin, while sensitive, is not specific to eosinophil-rich inflammation. Eosinophil-related biomarkers such as ECP might provide additional information, especially given evidence that eosinophils are abundant in IBD tissues. Previous research has proven an increase in numbers of activated and degranulating eosinophils in the colonic mucosa of IBD patients [4]. Even patients with silent UC can have a high density of activated eosinophils in their colon, and peripheral blood eosinophilia has been associated with worse clinical outcomes in UC. These observations suggest that eosinophil activity persists in IBD and may influence disease course.

Serum ECP has been investigated in IBD as a potential indicator of eosinophil-driven inflammation. Prior research reported that serum eosinophil levels are elevated in active disease compared to inactive disease and healthy controls [7], which might suggest ECP as a marker of disease activity in children [8]. In adults, fecal ECP levels are elevated in IBD and may even be increased in patients in clinical remission, especially in younger individuals [5]. Additionally, eosinophil granule proteins are increased locally in the gut during active IBD: for instance, ECP concentrations in intestinal lavage fluid are significantly higher in IBD patients than in controls [9]. This raises the question of whether ECP indicates subclinical mucosal inflammation or an “eosinophil-rich” inflammatory phenotype independent of clinical activity. Recent evidence suggests that IBD patients with eosinophil-predominant colonic inflammation might follow a different clinical course than those with neutrophil-dominant inflammation. For example, eosinophil-predominant IBD has been associated with fewer flares and hospitalizations compared to neutrophil-predominant disease, highlighting that the role of eosinophils in IBD is complex and may not simply mirror conventional markers of disease severity [1].

Despite these insights, ECP is not part of routine IBD assessment, and data on its usefulness in adult IBD are limited. The present study was undertaken to underline the possible usefulness of serum ECP in IBD. We aimed to measure serum ECP in adults with IBD and healthy controls. The primary objective was to compare serum ECP across UC, Crohn’s disease, and controls, with UC vs controls as the prespecified primary endpoint. Secondary objectives were to test associations between ECP and clinical activity (CDAI in CD; Mayo in UC) and patient-reported quality of life (IBDQ). Exploratory objectives were to examine correlations between ECP and hematologic indices (white blood cell, neutrophil, and eosinophil counts) and to explore the discrimination of UC vs HC. We hypothesized that ECP would be highest in UC, intermediate in CD, and lowest in HC and that ECP would correlate positively with leukocyte and neutrophil counts.

## Materials and methods

Study design and population

We performed a descriptive cross-sectional study on adult patients diagnosed with IBD (UC or Crohn’s disease) at a single tertiary gastroenterology center, using consecutive sampling. Patients were eligible if they had an established IBD diagnosis based on ECCO guidelines criteria, integrating clinical, endoscopic, histologic and imaging findings [10]. We included patients irrespective of disease activity status in order to capture a range of clinical scenarios. Exclusion criteria were significant concomitant conditions that could affect eosinophil counts or ECP levels (such as parasitic infections, hypereosinophilic syndromes, pregnancy or active allergic diseases), and use of systemic corticosteroids in the month prior to sampling (to avoid acute effects on eosinophil counts). We recorded 5-ASA, thiopurines, biologics, corticosteroids (dose and timing), antibiotics, inhaled/intranasal steroids, antihistamines and recent changes (less than eight weeks). A group of healthy individuals without IBD or other inflammatory or gastroenterological conditions was recruited as a control group, being recruited from hospital staff or community volunteers, they were screened by brief history and questionnaire to exclude gastrointestinal disease, current allergic disease (asthma, allergic rhinitis, atopic dermatitis), recent acute infection (less than four weeks), parasitic disease and chronic inflammatory conditions. In total, 50 IBD patients (20 with Crohn’s disease and 30 with UC) and 32 controls were included. The control subjects were similar to the IBD patients in age and sex distribution (59% female in controls vs 50% in Crohn’s and 40% in UC, p = 0.28 for sex difference). All participants provided written informed consent. The study protocol was approved by the institutional ethics committee, and the research was conducted in accordance with the principles of the Helsinki Declaration. 

Data collection

For each participant, we recorded demographic information, and clinical disease activity was assessed using the validated disease-specific indices: the Crohn’s Disease Activity Index (CDAI) and the full Mayo score for UC. These indices were calculated based on the standard criteria (including symptoms, examination findings and laboratory results) on the day of sampling. Patients were further categorized as being in clinical remission or active disease according to these scores (for example, CDAI <150 defined as remission in Crohn’s disease, and Mayo score ≤2 defined as remission in UC). We aligned assessments on the same day where feasible. Allowed windows were ± 7 days between blood draw, endoscopy and CDAI/MAYO/IBDQ. As a measure of patient-reported outcome, we administered the validated Inflammatory Bowel Disease Questionnaire (IBDQ) to IBD patients, yielding a total IBDQ score (higher scores indicating better quality of life). Use of the Inflammatory Bowel Disease Questionnaire, authored by Dr. Jan Irvine et. al., was made under license from McMaster University, Hamilton, Canada [11].

Blood samples were obtained from all IBD patients for laboratory analyses. We measured complete blood count parameters including hemoglobin (Hb), hematocrit (Ht), total white blood cell count (WBC) and differential counts (with particular interest in absolute eosinophil count, “Eo”, and neutrophils, “Neu”), and platelet count (PLT). 

Laboratory measurements and statistics

Fasting blood samples were collected in the morning (after an overnight fast of at least 10 hours) to measure serum ECP. Samples were collected into gold-cap serum separator tubes, centrifuged at 3000g for 10 minutes at room temperature within 60 minutes of draw, and the resulting serum was aliquoted into cryovials and frozen at -80 °C within 90 minutes. There was a maximum of one freeze-thaw prior to assay. Samples with gross hemolysis or lipemia were excluded after visual examination. Samples were stored for a maximum of 12 months. ECP was measured in batch using a commercial enzyme-linked immunosorbent assay (ELISA) kit (Elabscience®, Elabscience Bionovation, USA, catalog no. E-EL-H1379, lot PA1620LZ7012), with processing carried out according to the manufacturer’s instructions. The ELISA plates were read using an automated microplate reader (TECAN Sunrise™, Tecan Trading AG, Switzerland). In parallel, a complete blood count, including eosinophil counts, was performed using an automated hematology analyzer (Sysmex XN-550, Sysmex Corporation, Japan), according to standard laboratory procedures, under daily internal quality check. Laboratory personnel were blinded to groups, disease activity, and IBDQ scores. 

Statistical analysis was carried out using RStudio Desktop (RStudio©, PBC v1.4.1106, Boston, MA, USA). Analysis of normality was performed for all quantitative variables using the Shapiro-Wilk test; for parameters not normally distributed, non-parametric tests were applied. Comparison of medians between groups was done using the Kruskal-Wallis test and the Mann-Whitney U test with post hoc analysis using Dunn’s test. Correlations were analyzed using Spearman’s correlation coefficient. A value of p<0.05 was considered significant for all analyses.

Ethical considerations

The study protocol was approved by the institutional ethics committee (No. Ad. 11067 from 19.05.2023) and was conducted in accordance with the Declaration of Helsinki. All participants provided written informed consent prior to inclusion and blood sampling. The study involved no experimental interventions, and patient management was not altered by this research. Data were de-identified to protect patient confidentiality.

## Results

Characteristics of study subjects

A total of 50 IBD patients (median age 40 years, range 19-68; 46% female) and 32 healthy controls (median age 38 years, range 21-60; 59% female) were analyzed. Among the IBD patients, 20 had Crohn’s disease and 30 had UC. The Crohn’s disease group had a median age of 42 years (range 19-65), with 50% female patients, and the UC group had a median age of 38 years (range 22-68), with 40% female patients. The control group did not differ significantly from the IBD patients in terms of age or sex distribution. The median disease duration was six years (IQR 4-11.5) for Crohn’s disease patients and 6.5 years (IQR 2-14) for UC patients (no significant difference, p = 0.89). At the time of evaluation, 14 Crohn’s patients (70%) were in clinical remission (CDAI < 150) and 6 (30%) had mildly to moderately active disease. In the UC cohort, 18 patients (60%) were in remission (Mayo score 0-1) and 12 (40%) had active disease of at least mild severity. These proportions reflect a real-world outpatient sample with a mix of disease activity states. The median IBDQ score was somewhat lower (indicating worse quality of life) in Crohn’s patients compared to UC patients (164.5 (IQR 109-196) vs 189.5 (149-213)), but this difference did not reach statistical significance (p = 0.104). Tables 1, 2 summarize key baseline characteristics of the study groups.

**Table 1:** Serum ECP levels among controls, Crohn’s disease, and ulcerative colitis patients This table compares serum eosinophilic cationic protein (ECP) concentrations, expressed as medians with interquartile ranges (IQR), across controls (n=32), Crohn’s disease (n=20), and ulcerative colitis (n=30). P-values were obtained using Kruskal-Wallis with Dunn’s post-hoc test. Ulcerative colitis patients had significantly higher serum ECP compared to controls (p = 0.008), while Crohn’s disease patients did not differ significantly. Pairwise comparisons are also shown.

Variable	Control (n=32)	Crohn’s Disease (n=20)	Ulcerative Colitis (n=30)	p value (overall)
Female sex, n (%)	19 (59%)	10 (50%)	12 (40%)	0.28
Serum ECP in pg/mL (IQR)	16,807 (13,007-24,314)	18,496 (12,261-28,231)	24,224 (17,335-32,997)	0.03
– ECP vs Control p-value	–	0,418	0,008	–
– ECP vs Crohn’s p-value	–	–	0,128	–

Serum ECP levels in IBD vs. controls

**Figure 1:**
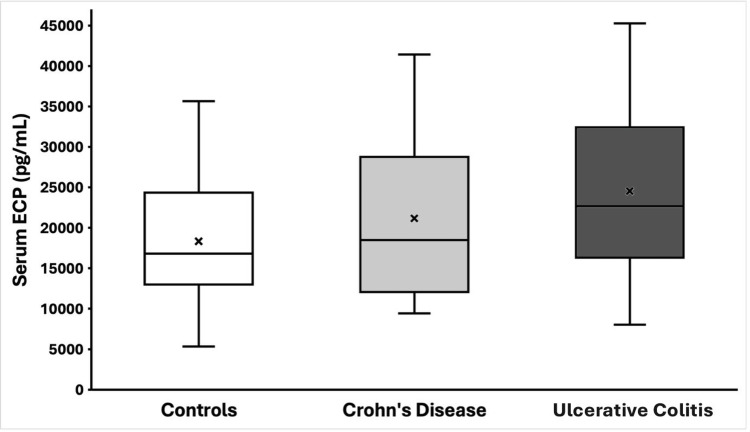
Serum ECP levels in healthy controls vs IBD patients Box-and-whisker plots illustrating serum eosinophilic cationic protein (ECP) concentrations (pg/mL) in healthy controls (n=32), Crohn’s disease (n=20), and ulcerative colitis (n=30). Boxes show interquartile ranges, horizontal lines indicate medians, whiskers represent ranges, and “×” marks group means. Statistical analysis was performed using Kruskal-Wallis with Dunn’s post-hoc test. Serum ECP was significantly higher in ulcerative colitis compared to controls (p = 0.008). Crohn’s disease patients showed a non-significant increase compared to controls. IBD: Inflammatory bowel disease

Serum ECP was detectable in all subjects and showed considerable inter-individual variability. The distribution of ECP values for each group is illustrated in Figure 1. IBD patients tended to have higher ECP levels than healthy controls. The median ECP concentration in the control group was 16,807 pg/mL (IQR 13,007-24,314 pg/mL). In Crohn’s disease patients, the median ECP was 18,496 pg/mL (IQR 12,261-28,231), while UC patients had a median ECP of 24,224 pg/mL (IQR 17,335-32,997). The Kruskal-Wallis test indicated a significant difference in ECP among the three groups (p = 0.03). Post hoc paired comparisons revealed that UC patients had significantly higher serum ECP than controls (Dunn’s test p = 0.008). The Crohn’s disease group also showed a higher median ECP than controls, but this difference was not statistically significant (Dunn’s p = 0.418). When comparing the two IBD subgroups, UC patients had higher ECP levels than Crohn’s patients, even if the difference did not reach significance (p = 0.128). The elevation of ECP was most pronounced in the UC cohort. It is notable that 60% of UC patients had ECP values above the upper quartile of the control range, whereas only 35% of Crohn’s patients exceeded that threshold. These findings indicate that ECP release is generally increased in IBD, particularly in UC. 

Comparison of Crohn’s disease vs. ulcerative colitis clinical and laboratory parameters

Aside from ECP, we compared other clinical and laboratory measures between the Crohn’s disease and UC groups (Table 2). The median total white blood cell count was slightly higher in Crohn’s patients (10.0×10^3/µL, IQR 6.59-11.93) than in ulcerative colitis patients (7.23×10^3/µL, IQR 6.03-9.26), but this difference was not statistically significant (p = 0.766). Differential counts showed that neutrophils were the major fraction of WBC in both groups, and the median absolute eosinophil counts were similar: 0.105×10^3/µL (IQR 0.07-0.29) in Crohn’s vs 0.145×10^3/µL (0.08-0.27) in UC (Mann-Whitney U p = 0.62). These values are within normal limits, indicating that most patients did not have marked peripheral eosinophilia at the time of sampling. Hemoglobin levels, hematocrit, and platelet counts did not differ significantly between Crohn’s and UC groups (all p > 0.2, data not shown). As mentioned, the disease duration was nearly identical between the two IBD groups (median ~6 years in each, p = 0.89).

**Table 2:** Laboratory and clinical comparisons between Crohn’s disease and ulcerative colitis patients This table presents median values with IQR for white blood cell counts, absolute blood eosinophil counts, disease duration, and quality of life scores (IBDQ) in Crohn’s disease versus ulcerative colitis. Use of the IBDQ was made under license from McMaster University, Hamilton, Canada. Statistical comparisons were performed using the Mann-Whitney U test. No significant differences were observed between the two groups. IBDQ: Inflammatory Bowel Disease Questionnaire

Variable	Crohn’s Disease	Ulcerative Colitis	p-value
WBC (×10^3/µL) (IQR)	10 (6.59-9.93)	7.23 (6.03-9.26)	0.766
Blood eosinophils (×10^3/µL) (IQR)	0.105 (0.07-0.29)	0.145 (0.08-0.27)	0.62
Disease duration (IQR)	6 (4-11.5)	6.5 (2-14)	0.89
IBDQ score (IQR)	164.5 (109-196)	189.5 (149-213)	0.104

Correlation of ECP with disease activity and other parameters

We next examined if serum ECP levels were associated with indices of disease activity, inflammation, or patient-reported outcomes. For this analysis, we considered the IBD patients as a single combined group (n = 50) and calculated Spearman correlation coefficients between ECP concentration and various continuous variables (Table 3). Serum ECP showed a weak-to-moderate positive correlation with the total white blood cell count (r = 0.279, p = 0.049). A similar correlation magnitude was observed between ECP and neutrophil count (r = 0.298, p = 0.036). These correlations suggest that higher ECP levels tend to accompany higher leukocyte counts. In contrast, there was no significant correlation between ECP and the absolute eosinophil count in peripheral blood (r = 0.207, p = 0.149). Interestingly, some patients with normal eosinophil counts had very elevated ECP, implying that ECP may reflect eosinophil activation rather than number. We did not find a significant correlation between ECP and disease activity scores. In the Crohn’s disease subset, the correlation between ECP and CDAI was negligible (r = 0.112, p = 0.638). In the ulcerative colitis subset, ECP also did not correlate with the total Mayo score (r = 0.087, p = 0.644). Even focusing on the Mayo score (available for those who had recent endoscopy), we did not observe a clear pattern (for example, patients with endoscopic remission could have either low or high ECP). There was likewise no significant correlation between ECP and the IBDQ quality of life score (r = 0.024, p = 0.865). ECP also showed no meaningful association with patient age (r = 0.051, p = 0.721) or with the duration of IBD (r = 0.22, p = 0.125).

**Table 3:** Spearman correlations between serum ECP levels and clinical variables in IBD patients This table shows Spearman correlation coefficients (r) and p-values for associations between serum ECP and demographic, clinical, and laboratory parameters in IBD patients (n=50). ECP correlated positively with total white blood cell and neutrophil counts, but not with disease activity indices (CDAI, Mayo), IBDQ score, or eosinophil counts. IBDQ: Inflammatory Bowel Disease Questionnaire; IBD: inflammatory bowel disease; ECP: eosinophilic cationic protein

Variable Compared to ECP Levels	Spearman r	p-value
Age (years)	0.051	0.721
Disease duration (years since diagnosis)	0.22	0.125
Crohn’s Disease Activity Index (CDAI) – CD subset	0.112	0.638
Mayo score – UC subset	0.087	0.644
IBDQ score	0.024	0.865
White blood cell count (WBC)	0.279	0.049
Neutrophil count	0.298	0.036
Eosinophil count (absolute)	0.207	0.149
Hemoglobin	0.111	0.442
Hematocrit	0.161	0.263
Platelet count	0.142	0.324

In summary, our results indicate that while serum ECP is elevated in IBD (especially UC) compared to healthy controls, within our patient cohort ECP did not track linearly with the standard clinical activity indices or quality of life measures. The only significant associations for ECP were with general blood leukocyte counts, hinting that ECP may rise in parallel with overall inflammatory cell activity.

## Discussion

In this observational study, we investigated the role of ECP as a biomarker in IBD. We found that serum ECP levels were higher in IBD patients than in healthy controls, with the difference driven largely by patients with UC. UC patients showed a marked elevation in ECP relative to controls, whereas Crohn’s disease patients had a more modest increase that did not reach statistical significance. This pattern aligns with the known histopathological features of IBD: UC often involves robust eosinophilic infiltration in the colonic mucosa, sometimes exceeding that seen in Crohn’s disease. This finding supports the idea that eosinophil activation is a prominent component of UC inflammation, potentially more so than in Crohn’s disease. In fact, prior tissue studies have reported higher densities of activated or degranulating eosinophils in UC colon biopsies, which could explain the higher systemic ECP levels in these patients. Notably, even some UC patients in clinical remission exhibited elevated ECP in our study, echoing observations that eosinophils can remain active in mucosal repair or smoldering inflammation during quiescent disease [4].

Another important result of our study is that serum ECP correlated significantly with the total leukocyte and neutrophil counts. This suggests that ECP increases in tandem with general inflammatory activity. High WBC and neutrophil counts typically reflect active inflammation or infection; the positive correlation with ECP implies that when the body’s overall inflammatory response is heightened (as indicated by leukocytosis), eosinophils are likely contributing and releasing ECP as part of that response. However, we did not find a correlation between ECP and blood eosinophil count. This lack of correlation indicates that the amount of ECP in the circulation is not simply a function of how many eosinophils are present in blood, but rather how activated they are and possibly the extent of eosinophil activity in tissues. Eosinophils can secrete ECP when stimulated and can do so within tissues without a marked peripheral eosinophilia. Thus, a patient could have normal eosinophil counts but elevated ECP if tissue eosinophils are actively degranulating. Our findings are in line with this concept, and similar phenomena have been noted in allergic conditions: for example, serum ECP often correlates poorly with eosinophil count in asthma, yet it reflects disease activity. In the context of IBD, this implies ECP might capture an aspect of inflammation not visible by routine blood counts.

Contrary to our expectations, serum ECP did not show a significant correlation with clinical disease activity indices (CDAI in Crohn’s or Mayo score in UC) in our adult patient cohort. There are a few possible explanations for this. First, our study sample included a substantial proportion of patients in remission or with mild disease, which restricted the range of activity scores and may have limited our power to detect correlations. The CDAI and Mayo scores are also imperfect measures; they incorporate subjective symptoms and, in the case of CDAI, can be influenced by factors like irritable bowel symptoms even when inflammation is low. It is conceivable that eosinophil-related inflammation (as reflected by ECP) might not fluctuate in parallel with those indices, especially in patients whose disease is primarily neutrophil-driven or who have localized disease activity. In pediatric studies, however, the situation appeared different: Wedrychowicz et al. reported moderate correlations between serum ECP and pediatric activity indices (R ~0.5) and showed that ECP levels decrease with effective therapy [8]. Our inability to demonstrate such correlations in adults might be due to differences in study design (cross-sectional vs longitudinal) or population. The pediatric study measured ECP at diagnosis and then longitudinally during treatment, capturing dynamic changes. By contrast, our cross-sectional design captured single time-point measurements; some patients in remission had elevated ECP from previous inflammation or ongoing subclinical inflammation, which did not mirror their clinical score at that moment. Indeed, one UC patient in clinical remission in our cohort had an ECP level of ~35,000 pg/mL, possibly indicating residual microscopic inflammation not accounted for by the clinical remission status. This highlights that ECP might be detecting inflammatory activity that clinical indices or even endoscopy miss. It has been shown that mucosal healing can lag behind symptomatic improvement, and conversely, subtle inflammation can persist in clinically quiescent IBD. ECP might be a marker that flags this subtle ongoing inflammation. Supporting this idea, a study by Yamamoto-Furusho et al. demonstrated that eosinophils in UC can be activated and release granule proteins even when the disease is in remission endoscopically [12]. Research has also shown that eosinophils can be locally activated and degranulate in response to various cytokines, such as IL-33, which promotes eosinophil adhesion and secretion of granule constituents like eosinophil-derived neurotoxin [13]. This suggests that the inflammatory environment in UC may remain conducive to eosinophilic activity despite the absence of overt disease symptoms. Additionally, the presence of eosinophils in colonic tissues correlates with disease severity and may indicate a histopathological persistence of inflammation, as eosinophil numbers can increase during prolonged phases of the disease, potentially impacting long-term disease management [14,15].

Our findings are broadly consistent with earlier investigations into eosinophil granule proteins in IBD. Troncone et al. first showed that children with IBD have significantly higher levels of ECP in gut lavage fluid compared to controls, suggesting that eosinophils in the gut are actively degranulating in IBD. We extend that knowledge by showing a systemic elevation of ECP in adult IBD, particularly UC [9]. Pronk-Admiraal et al. focused on UC and found that serum ECP was higher in active disease than in quiescent disease. They also noted that as patients were treated and went into remission, ECP levels declined in parallel with decreases in blood eosinophil counts and CRP [16]. Our study did not include longitudinal follow-up, but the cross-sectional differences we observed between active and inactive subgroups (though not statistically robust) hint at a similar trend: active UC patients in our sample had numerically higher ECP than those in remission. It would have been informative to follow our patients over time to confirm the responsiveness of ECP to treatment; we propose this as a direction for future research.

 Beyond serving as an activity marker, ECP and eosinophils in general may have pathophysiological significance in IBD. ECP is a toxic cationic protein capable of damaging epithelial cells and impairing barrier function. Experimentally, neutralization of ECP has been shown to reduce colitis severity in animal models: Shichijo et al. demonstrated that administering antibodies against ECP ameliorated colonic inflammation in a dextran sulfate sodium rat model of colitis [17]. This finding implies that ECP is not just a bystander marker but likely contributes to mucosal injury in IBD. Eosinophils also release other granule proteins (such as eosinophil peroxidase, major basic protein, and eosinophil-derived neurotoxin) which can all cause tissue damage or modulate inflammation. The collective action of these eosinophil products may promote a Th2-type inflammatory milieu, especially in UC which has some features of Th2 immune response. Interestingly, blocking eosinophil recruitment or survival has shown promise in related diseases (e.g., anti-IL-5 therapy in eosinophilic gastrointestinal disorders). In IBD, therapies targeting eosinophils are not yet in clinical use, but there is growing interest in whether a subset of IBD patients (those with eosinophil-predominant inflammation) might benefit from such strategies. Our work underlines that identifying those patients is possible through biomarkers like ECP or tissue eosinophil counts [4].

It is also worth discussing the potential of ECP as a predictive biomarker. Recent research by Abedin et al. examined fecal ECP in young adults with IBD and found it to be a useful diagnostic and predictive biomarker [5]. High fecal ECP was associated with a higher risk of relapse, indicating that eosinophil-driven inflammation could herald disease exacerbation. While our study assessed serum ECP rather than fecal, one might expect that fecal ECP (originating directly from intestinal eosinophils) could be even more closely related to mucosal inflammation. Some of our UC patients in remission but with elevated serum ECP later experienced minor flare-ups (according to follow-up visits outside the scope of this study), which is anecdotal evidence aligning with the notion that elevated ECP might precede clinical relapse. However, a formal prospective study is needed to validate ECP’s predictive value. If confirmed, ECP could join fecal calprotectin as part of a panel to monitor IBD patients in remission for early signs of recurrence.

Our study has several strengths, including the use of a control group and the evaluation of ECP alongside detailed clinical indices and patient-reported outcomes. It provides a comprehensive look at where ECP fits among other measures. Nonetheless, we must acknowledge important limitations. The sample size, especially when divided into subgroups, was relatively small, which may have led to type II errors (failing to detect some true correlations or differences). The cross-sectional design limits causal interpretations; we cannot be certain whether high ECP is a cause or consequence of more severe disease. Also, the single-center tertiary setting and consecutive sampling may limit external validity. Additionally, we did not measure other inflammatory markers (like CRP, fecal calprotectin) at the exact same time point for all patients, which could have provided context on how ECP compares or adds to those markers. We also note that the ECP assay we used, while standard, reports values in pg/mL; other studies use ng/mL, so one must be careful in comparing absolute values between studies (1 ng/mL = 1000 pg/mL). All our groups had median ECP values in the tens of ng/mL range, consistent with previously reported levels.

Despite these limitations, our findings add to the growing evidence that eosinophils and their products merit attention in IBD. They reinforce that UC is an eosinophil-rich disease and suggest that serum ECP could be a convenient surrogate for that aspect of inflammation. Given our cross-sectional case-control design, well suited to detecting between-group differences and simple correlations, we cannot infer causality, assess within-patient responsiveness to therapy, or determine predictive value; accordingly, we refrain from claims about subclinical inflammation or relapse prediction and interpret our results as descriptive and hypothesis-generating. We recognize that current medications, smoking, BMI, atopy/seasonality, and disease extent can influence both leukocyte profiles and ECP; however, because the study is small and cross-sectional and some of these factors may lie on the causal pathway, we did not fit multivariable models to avoid overfitting and over-adjustment. Residual confounding is therefore not impossible, and all findings should be interpreted with appropriate caution. For clinicians, measuring ECP might have a role in special scenarios; for example, in a UC patient who reports feeling well but has an equivocal endoscopic finding, an elevated ECP might prompt a closer look for occult inflammation. Conversely, a normal ECP in a patient with mild symptoms could reassure that eosinophil-mediated inflammation is minimal (though neutrophil inflammation could still be present). These are speculative applications, and more research is needed to substantiate them.

## Conclusions

Our study demonstrates that ECP is a potentially useful biomarker in IBD, especially for UC. Specifically, we conclude that serum ECP is significantly elevated in IBD patients compared to healthy controls, with the highest levels observed in UC. This highlights the involvement of eosinophils in the inflammatory process of IBD, particularly in colonic disease. Also, serum ECP correlates with general markers of inflammation (WBC and neutrophil counts) but not with standard clinical activity indices in our cohort. This suggests ECP reflects an inflammatory component that may not directly translate into symptom-based scores, possibly subclinical or tissue-level eosinophil activity. ECP shows preliminary promise as a complementary marker, pending validation. Monitoring ECP could help identify ongoing mucosal inflammation or predict relapse, especially in patients with UC. However, its current clinical utility is limited by the need for further documentation.

Looking forward, larger prospective studies should evaluate how ECP levels change with treatment and whether they predict long-term outcomes (e.g., flares, response to therapy, need for surgery). It would also be valuable to study fecal ECP in parallel with serum ECP to determine which is more informative and practical. Given that targeted anti-eosinophil therapies are under consideration in related disorders, identifying an eosinophil-rich endotype of IBD is an important task, and ECP could be one marker to define that endotype. In summary, our findings contribute to the understanding of eosinophil involvement in IBD and lay the groundwork for considering ECP as part of a multifaceted approach to IBD biomarker development.
